# Vaccine Hesitancy: Characteristics of the Refusal of Childhood Vaccination in a Peruvian Population

**DOI:** 10.7759/cureus.14105

**Published:** 2021-03-25

**Authors:** Kocfa Chung-Delgado, Javier E Valdivia Venero, Tuong M Vu

**Affiliations:** 1 School of Health and Related Research, University of Sheffield, Sheffield, GBR; 2 Faculty of Medicine, Universidad Peruana de Ciencias Aplicadas, Lima, PER

**Keywords:** vaccine, immunization, vaccine hesitancy, pacv, peru, vaccine refusal

## Abstract

Understanding the determinants of vaccine hesitancy is paramount to reinstate confidence in immunizations. The objective of this investigation was to explore the characteristics of the vaccination decision-making process that may result in the refusal of childhood immunization in Peru, during February-June 2020. A descriptive, cross-sectional study involving telephone interviews was executed in Peru. The Parents Attitudes about Childhood Vaccines (PACV) survey was used. A demographic analysis was done, followed by an unadjusted exploratory subgroup analysis. Out of 552 subjects, 9.8% were considered vaccine hesitant, 70.3% had purposively delayed vaccination, 88.4% thought fewer vaccines were better and 52.2% were concerned about vaccine safety. The level of hesitancy was inversely proportional to the level of education and the number of children at home. Mothers and subjects aged ≤29 years showed a greater level of vaccine hesitancy. This population displays a vaccine-hesitant conduct. Vaccine safety and the number of vaccines to administer are important determining factors. This behavior could be influenced by variables such as level of education, number of children at home, parental relationship, and age. These results help understand local vaccination behaviors. More studies are encouraged to confirm and validate these findings.

## Introduction

Vaccines have greatly contributed to the control and eradication of diseases around the world [1]. Despite plentiful scientific evidence validating the use of vaccines, some parties have challenged this credibility and deemed vaccines as unsafe and unnecessary. Individuals or groups that display a defiant conduct against vaccines are called anti-vaccines, vaccine refusers, or anti-vaxers [2]. Moreover, a new term was coined to refer to those who were neither passionate advocates nor radical protesters of vaccines, being in the middle portion of the acceptance spectrum: vaccine hesitancy [3].

The World Health Organization’s (WHO) Strategic Advisory Group of Experts on Immunization (SAGE) defines individuals with vaccine hesitancy as those who “…delay the acceptance or refusal of vaccines despite availability of vaccines services” [3]. They are essentially, a group of individuals with forgiving doubt and skepticism, yet not rejection. As a behavior, vaccine hesitancy is driven by numerous factors and conditions. Vaccine hesitancy has hindered historic public health efforts [2].

Understanding the true reasons leading to vaccine hesitancy is paramount to reinstate confidence in immunizations. Currently, most of the evidence depicting vaccine hesitancy comes from developed countries, therefore limiting the extrapolation that can be done in the context of developing economies. This is the case for Peru [4], a country that is beginning to see the effects of vaccine hesitancy in its population. In accordance with this important scientific gap, the aim of this research was to investigate the characteristics of the vaccination decision-making process that may result in the refusal of childhood immunization in Peru.

## Materials and methods

The objective of this study was to investigate the characteristics of the vaccination decision-making process that may result in the refusal of childhood immunization in parents from Lima, Peru, during the months of February-June 2020.

A descriptive, interview-based, cross-sectional study was executed. It was performed via a structured telephone interview with subjects recruited from a pediatric private practice office in the northern metropolitan region of Lima, Peru. This region was selected due to the high concentration of healthcare services and providers, therefore reducing the probability of vaccine-related systematic or access failures [4]. The questionnaire used was the validated Parent Attitudes about Childhood Vaccines (PACV) survey, Spanish version [5-7].

Sampling and data collection

Subjects were initially identified through convenience purposive sampling by the pediatric physician at the private practice office. Once identified according to the inclusion and exclusion criteria, the physician invited the subject to participate in the study by reviewing the Informed Consent Form (ICF) and the Patient Information Sheet (PIS). If agreed upon and the ICF signed, the physician registered the subject in a database and the subject was later contacted via telephone to complete the questionnaire interview.

The inclusion criteria were as follows: Spanish-speaking subjects 18 years or older, subjects who attended the pediatric consult regarding a childhood immunization topic (vaccinating clinic, information request, immunization scheduling, vaccination cards, etc.), being the parent or legal guardian of the child with regard to the immunization topic, and accepting the informed consent.

The exclusion criteria were as follows: withdrawal of informed consent at any part during the study, incomplete responses in the PACV survey, and inability to be reached after three telephone contact attempts.

Parent Attitudes about Childhood Vaccines survey

The PACV is a 23-item questionnaire that was created based on the WHO’s Determinants of Vaccine Hesitancy Matrix. It combines general demographic questions and vaccine-hesitant behavior questions, either in a linear numeric scale or a five-point Likert scale format [5]. The results of the PACV are calculated based on a 30-point system and later converted into a 0-100% score [5,8]. To maintain the external validity of the survey, the same score categorization from the original validation study was used: 0-50%, vaccine-compliant parents; 51%-69%, hesitant parents with children who were immunized 8.3% more days late; and 70%-100%, hesitant parents with children who were immunized 46.8% more days late [8].

Sample size calculation

The sample size calculation was done through the PASS® software (NCSS LLC Statistics, UT, USA). A modified version of the Cochrane formula for sample size was applied since the distribution of vaccine hesitancy in the population was unknown. Higher rates of participation refusal and drop-outs were considered to account for the limitations of a telephone-based interview methodology [9]. Considering a 30% participation refusal rate and a 17% drop-out rate, assuming a confidence interval of 95% and a power of 80%, a total of 532 subjects needed to be included in the study [9].

Statistical analysis

Data and calculations were registered in a single database using a standard spreadsheet software (Microsoft Excel®; Microsoft, WA, USA). A double-entry process was followed to minimize human error in data translation.

All statistical calculations and analysis were done using this database. First, a demographic analysis was performed to understand the profile of the subjects. Then, the individual PACV items were analysed to determine the overall proportion of subjects within the hesitancy spectrum. Subsequently, a secondary exploratory analysis was made to describe how these results varied according to variable subgroups. The four subgroups were based on the following variables: level of education, number of children in the household, relationship to the child, and age.

Ethical considerations

This study was conducted in accordance with Good Clinical Practice and the Declaration of Helsinki. The study protocol and supporting documentation were approved by University of Sheffield’s Ethics Review Board on February 18, 2020 (application reference #033003, document codes #1075724 and #1075727), United Kingdom. Furthermore, the study underwent an institutional review by the private pediatric practice where the subjects were recruited (approval reference LIM-DA-2020-02-24).

## Results

A total of 629 subjects were contacted during this study. Of those, 29 subjects (4.6%) refused to participate after being contacted. An additional 17 subjects (2.8%) dropped out during the interview. Of the remaining, 31 (5.3%) subjects were excepted due to exclusion criteria. Hence, a total of 552 subjects were included in the final analysis.

Of the total subjects included, 394 (71.4%) identified as the child’s mother. A total of 286 (51.8%) were aged 18-29 years and 271 (49.1%) reported having two or more children at home. The detailed demographics results can be seen in Table 1.

**Table 1:** Demographic Characteristics of the Interviewed Population, Peru 2020

Demographics	Total n=552
Firstborn child, n (%)	
Yes	312 (56.5%)
No	240 (43.5%)
Age, n (%)	
18-29 years	286 (51.8%)
≥30 years	266 (48.2%
Relationship to the child, n (%)	
Mother	394 (71.4%)
Father	115 (20.8%)
Other	43 (7.8%)
Marital status, n (%)	
Single	16 (2.9%)
Married	148 (26.8%)
Living with partner	206 (37.3%)
Widowed	9 (1.7%)
Separated	69 (12.5%)
Divorced	104 (18.8%)
Highest level of education, n (%)	
≤8th grade	28 (5.1%)
High school, non-graduate	87 (15.8%)
High school graduate	244 (44.2%)
College or a 2-year degree	102 (18.4%)
4-year degree	69 (12.5%)
More than a 4-year degree	22 (4.0%)
Number of children in the household, n (%)	
1	281 (50.9%)
2	171 (31.0%)
3	87 (15.8%)
≥4	13 (2.3%)
Declared ethnicity, n (%)	
White	41 (7.4%)
Black of African American	8 (1.4%)
Latino	489 (88.6%)
Asian	14 (2.6%)
Pacific Islander	0 (0%)
Alaska Native	0 (0%)
Other	0 (0%)

PACV scores and vaccine hesitancy 

Globally, 58.3% (n=322) of the subjects were in the non-hesitant part of the spectrum, while 230 (41.7%) of them were in the vaccine-hesitant pole. From this vaccine-hesitant pole pool, 23.5% (n=54; 9.8%) corresponded to the 70%-100% PACV category. The median was 49% and the maximum was reached at a PACV score of 94%.

A total of 70.3% (388) of the subjects had delayed the administration of a vaccine to a child for reasons other than illness or allergy. Similarly, 59.8% (330) of the subjects had decided not to administer a vaccine to a child for reasons other than illness or allergy. Regarding the safety of vaccines, 52.2% (288) of the subjects were “somewhat or very concerned” about the serious side effects of vaccines. Likewise, 48.7% (269) of them were concerned that the vaccine “might not be safe”.

Other results showed that 17.2% (95) of the subjects agreed that a child received too many shots. Furthermore, 88.4% (488) thought that fewer vaccines were better for children. Conversely, 81.7% (451) agreed that vaccines protect against very severe illnesses. Further details on other PACV results can be seen in Table 2.

**Table 2:** Results and Scores of the PACV Questionnaire, Peru 2020 PACV, Parent Attitudes about Childhood Vaccines

	Results (total n=552)				
PACV overall score, n (%)					
0-50% PACV category	322 (58.3%)				
51%-69% PACV category	176 (31.9%)				
70%-100% PACV category	54 (9.8%)				
Questions	Yes	Don’t know	No		
“Have you ever delayed having your child get a shot?”, n (%)	388 (70.3%)	37 (6.7%)	127 (23.0%)		
“Have you ever decided not to have your child get a shot?”, n (%)	208 (37.7%)	14 (2.5%)	330 (59.8%)		
“If you had another infant, would you want him/her to get all recommended shots?”, n (%)	462 (83.7%)	27 (4.9%)	63 (11.4%)		
Questions	Completely sure	Partially sure	Not at all sure		
“How sure are you about following the recommended shot schedule for your child?”, n (%)	152 (27.5%)	378 (68.5%)	22 (4.0%)		
“All things considered, how much do you trust your child’s doctor?”, n (%)	479 (86.8%)	55 (10.0%)	18 (3.3%)		
Questions	Very concerned	Somewhat concerned	Not sure	Not too concerned	Not at all concerned
“How concerned are you that your child might have a side effect?”, n (%)	94 (17.0%)	194 (35.2%)	104 (18.8%)	100 (18.1%)	60 (10.9%)
“How concerned are you that the shots might not be safe?”, n (%)	199 (36.0%)	70 (12.7%)	152 (27.5%)	76 (13.8%)	55 (10.0%)
“How concerned are you that the shot might not prevent the disease?”, n (%)	43 (7.8%)	88 (15.9%)	180 (32.6%)	129 (23.4%)	112 (20.3%)
Questions	Strongly agree	Agree	Not sure	Disagree	Strongly disagree
“Children get more shots than are good for them”, n (%)	11 (2.0%)	84 (15.2%)	401 (72.7%)	53 (9.6%)	3 (0.5%)
“It’s better for children to get fewer vaccines at the same time”, n (%)	467 (84.6%)	21 (3.8%)	52 (9.4%)	11 (2.0%)	1 (0.2%)
“I believe that many of the illnesses shots prevent are severe”, n (%)	407 (73.7%)	44 (8.0%)	92 (16.7%)	9 (1.6%)	0 (0.0%)
“It is better for my child to develop immunity by getting sick”, n (%)	15 (2.7%)	73 (13.2%)	148 (26.8%)	144 (26.1%)	172 (31.2%)
“I trust the information I receive about shots”, n (%)	102 (18.5%)	183 (33.1%)	245 (44.4%)	15 (2.7%)	7 (1.3%)
“I can openly discuss my concern about shots with the doctor”, n (%)	374 (67.8%)	88 (15.9%)	76 (13.8%)	14 (2.5%)	0 (0.0%)

Secondary exploratory analysis

**Figure 1:**
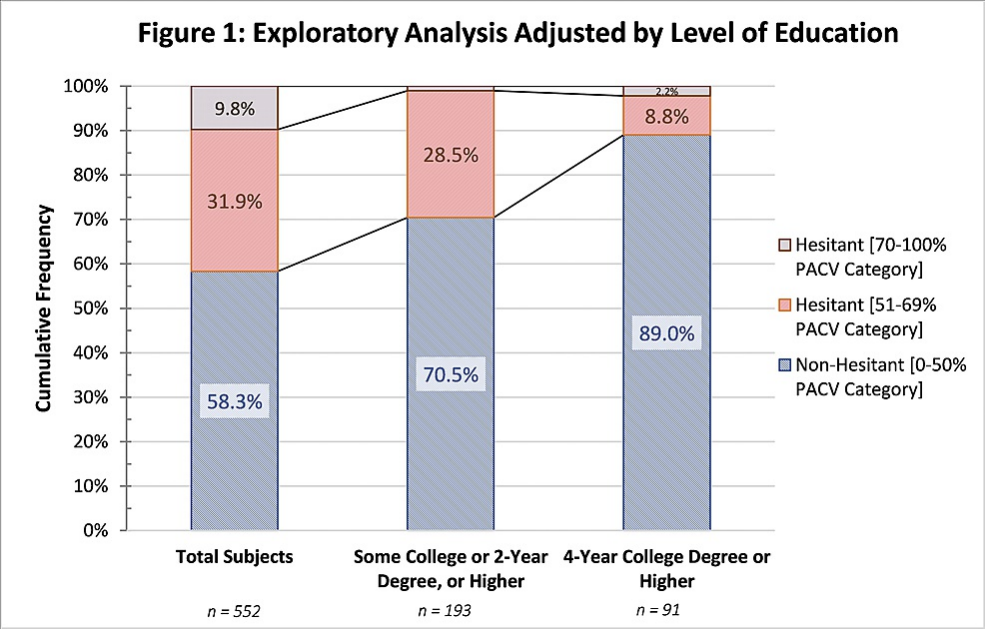
Exploratory Analysis Adjusted by Level of Education PACV, Parent Attitudes about Childhood Vaccines

The unadjusted calculation for the level of education including only those with “Some college or 2-year degree” or higher showed an increase of 12.2% in the non-hesitant category. Including only the “4-year college degree” and higher, the level of non-hesitant subjects rose even higher to 89.0% (81). A detailed transitional graph can be seen in Figure 1.

**Figure 2:**
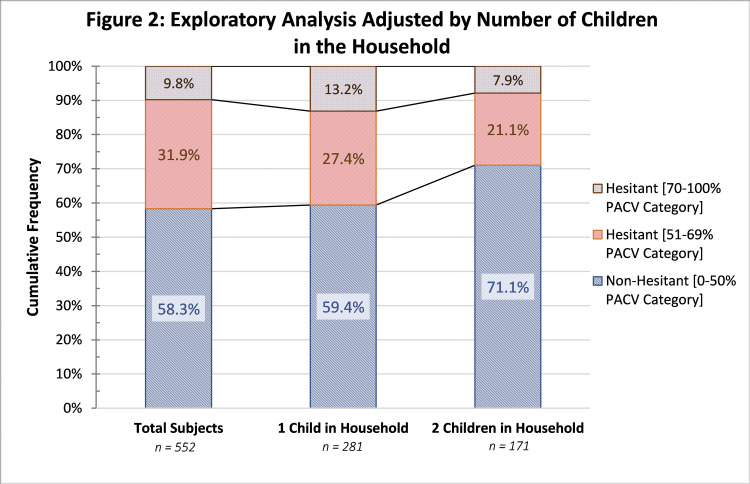
Exploratory Analysis Adjusted by Number of Children in the Household PACV, Parent Attitudes about Childhood Vaccines

The proportion of non-hesitant subjects increased to 71.1% (189) in families with two or more children in the household. An increase in hesitant subjects was seen in families with only one child in the household. More data can be seen in Figure 2.

**Figure 3:**
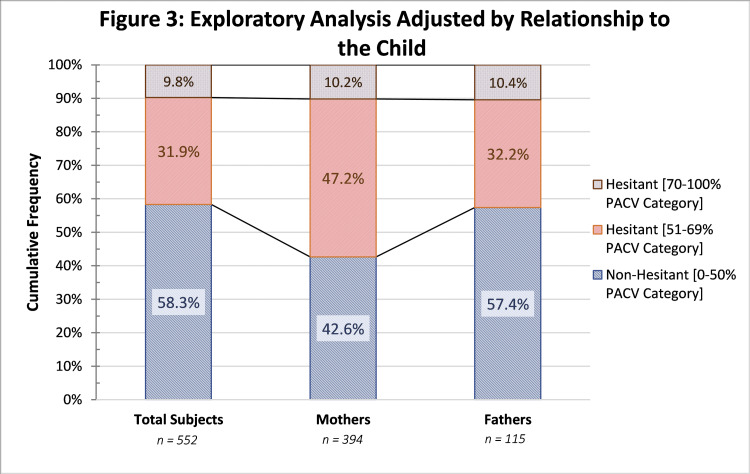
Exploratory Analysis Adjusted by Relationship to the Child PACV, Parent Attitudes about Childhood Vaccines

The hesitant proportion of subjects increased considerably when including mothers only. Less than 1% change was seen in the fathers-only group. The distribution can be seen in Figure 3.

**Figure 4:**
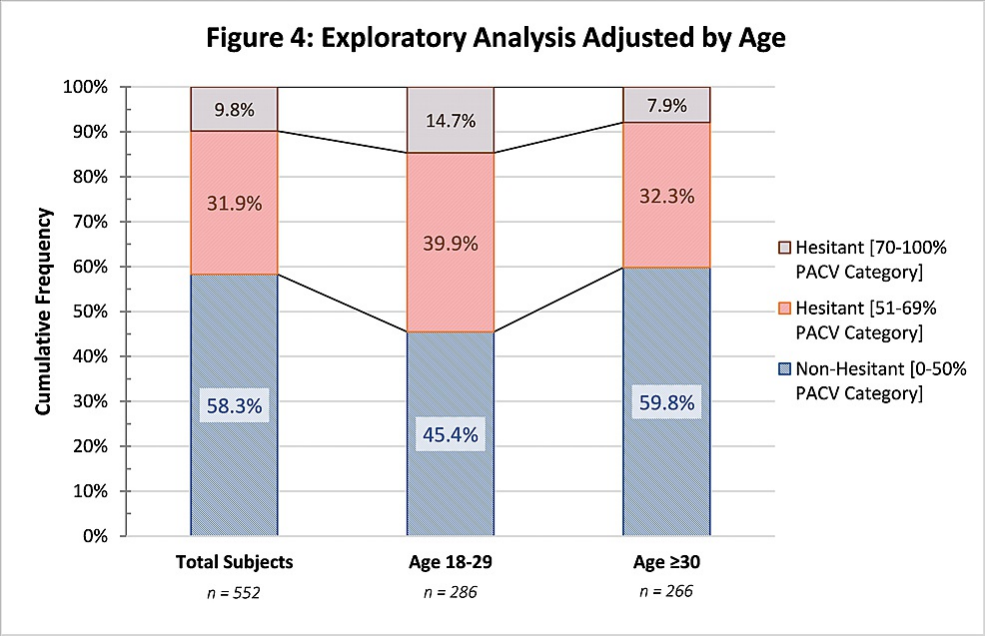
Exploratory Analysis Adjusted by Age PACV, Parent Attitudes about Childhood Vaccines

The number of non-hesitant subjects dropped to 45.4% for ages 18-29 but increased to 59.8% for ages ≥30. Notably, the proportion of hesitant subjects in the 70%-100% category group increased to 14.7% in the 18-29 age group. A detailed distribution by age can be seen in Figure 4.

## Discussion

As an overarching result, 58.3% of the subjects were considered non-hesitant towards vaccination. Most importantly, 9.8% of the subjects were on the hesitant pole of the spectrum, indicating strong doubts and uncertainty about vaccines. According to the original PACV validation, this means that these individuals are more likely to delay their children’s immunizations - to be more specific, at least 46.8% additional days [8].

This general result is aligned with other published investigations on the matter. A recent study by Raof found that 14.2% of parents were hesitant towards vaccines, considering them “late vaccinators” [10].

Knowing the prevalence of vaccine hesitant individuals is a valuable indicator towards patient education and immunization coverage. Evidence has shown that sharing information increases the uptake of vaccines and contributes to a positive vaccination coverage [11]. This is a key success factor when contributing to the strategic management of public immunization programs. Vaccine-hesitant individuals may warrant a greater need for scientific information to impart confidence and reassurance. The impact of such strategies has been described in previous studies as a successful intervention [11].

In this study, 70.3% of the population had delayed their child’s vaccination due to any reason other than illness or allergy. Comparatively, this proportion is higher than the results found in other studies: for example, Opel et al. concluded a delay in only 21.7% of the patients [12]. It is our conjecture that there is no strong reason to believe that this study has pinpointed an exceptional factor that led to an increased delay in vaccination in this Peruvian population. On that note, it is possible that the delay could have occurred due to one of many non-scientific reasons such as vaccine access, purchase price of vaccines, delayed appointments, vaccine supply, etc. This finding must be further investigated in order to clarify the true reasons behind this finding.

An undeniable reason that could have influenced this result is the severe acute respiratory syndrome coronavirus 2 (SARS-CoV-2) pandemic that was occurring in parallel to the execution of this investigation. By itself, the pandemic lockdowns are a compelling reason to delay vaccination, yet have no direct link to doubt or hesitancy towards vaccines. It is an example of a systematic determinant that influences but does not reflect vaccine hesitancy [3]. Given the context, this is an important confounder that must be taken into consideration as new evidence is highlighting the positive behavioral impact that the pandemic is having on vaccine-hesitant individuals.

Over 72% of the subjects referred that they were “not sure” about the quantity of vaccines administered to the child. This reflects a paramount behavior behind vaccine hesitancy as it is the factor with the greatest hesitancy proportion among all other results in this PACV survey. In addition, it is complemented with 84.6% of subjects who strongly agree that it is better for children to get fewer vaccines at the same time.

Scientific evidence on the matter has clearly determined that there is no “upper limit” on the number of vaccines that can be administered simultaneously [13]. During simultaneous administration, most vaccines maintain their efficacy and do not raise safety concerns in the patient [14].

The number of vaccines administered to a child, especially during the first year of age, has increased over the last 10 years. Literature related to the topic has concluded that “multiple immunizations have been, and could continue to be, of societal significance in terms of parental worries, potential health burdens, and future challenges for immunization policy-making” [15]. The findings of this study support this premise: unawareness of the safety of simultaneous vaccinations may contribute to the hesitancy in parents.

Vaccine safety is a factor known to fuel vaccine hesitancy in parents. McKee and Bohannon identified it as the probable greatest reason for vaccination delay [16]. In this study, 52.1% were “somewhat concerned” or “very concerned” about a side effect of the vaccine. A study conducted by Dubé et al. concluded that a common source of misinformation regarding vaccine safety was the mouth-to-mouth communication of clustered adverse events after immunization [17]. Additional factors linked with safety concerns are religious beliefs, long-term effects, ingredients in vaccines, among others [18].

Even though autism was not specifically referenced in this study, it has been acknowledged as one of the chief concerns in the long-term safety of vaccines and has impacted vaccine coverage [2]. Numerous studies and scientific evidence have discredited this false claim [19]. Locally speaking, Peru has seen this negative impact, indicating that this concern is of relevance to the local context [20].

Media communications and social media play an important role in the safety of vaccines [16]. Social media platforms have been identified as the single most aggravating factor towards avoiding vaccination [21]. Evidence suggests that viewing any anti-vaccine site for 5 to 10 minutes could be enough to increase the perception of risk of vaccines [21]. Even if sources are formatted in a pro-con fashion to depict a balanced view of the topic, the overall effect on readers may be predominantly negative, concluding that even balanced sources of media contribute to a negative impact and vaccine hesitancy overall [22].

As opposed to vaccine safety, vaccine efficacy did not demonstrate to be an utmost factor in vaccine hesitancy in this study. Regardless of this balance, having a 23.7% proportion of parents who are concerned with vaccine efficacy does raise an important issue.

Doubts about vaccine efficacy are more commonly linked to healthcare providers rather than patients [23]. A vaccine may have an efficacy below 100% and still be confidently efficacious [24]. For example, seasonal influenza vaccine efficacy is commonly found to be between 30% and 60%, whereas the measles-mumps-rubella vaccine efficacy is ≥95% [24]; nevertheless, this efficacy percentage does not discredit the value and benefit of the influenza vaccine, or any vaccine. It is important to note that the true value of vaccines should be perceived from a public health perspective too, and not solely at an individual level [24].

Another important finding in this study is that at least 81.7% of the subjects believe that the diseases that vaccines prevent are severe, and 57.3% of subjects disagree that it is better to develop immunity by getting sick.

As it is, the fact that individuals strongly believe that vaccines prevent severe diseases is a unique opportunity to counteract vaccine hesitancy. There is no need to counterargue or debate about its scientific accuracy, unlike vaccine safety or the quantity of vaccines administered. On the contrary, it can be used as a stepping stone to impart greater confidence in immunizations. The WHO has based many of its immunization policies on this element [1]. International scientific authorities also reference the disease severity as a leverage point for vaccine coverage and immunization programs [1].

Secondary exploratory analysis by variables

The secondary exploratory analysis demonstrated that some categorical values might have an effect on the overall vaccine hesitancy results. When the analysis was made by the level of education, a clear increase in vaccine-accepting individuals was seen. Considering only “Some college or 2-year degree” or higher, the non-hesitant group increased from 58.3% to 70.5%, as evidenced in Figure 1. In the same manner, the hesitant group dropped from 9.8% to 1.0%. On top of this, if the secondary analysis included only “4-year college degree” or higher, the vaccine-accepting group increased to 89.0%. This trend displays that the greater the education level, the greater the proportion of apparent acceptance towards vaccines.

In the published literature, the direct relationship between education level and vaccine acceptance remains unclear. Greater sources of information might allow patients for a more conscientious discernment and critical judgement of the data available. On the opposite hand, greater education induces broader questioning on vaccines. Investigations have identified that a higher level of education was related to a lower level of vaccine hesitancy [25]. Conversely, other investigations have concluded that greater education leads to a more critical point of view and thorough questioning of vaccines, noting that “higher levels of education were nearly four times as likely to be concerned about the safety of vaccines than those with lower education levels” [26]. In any case, it can only be concluded that the level of education has a confounder effect in the overall decision-making process of vaccine administration.

Based on this study, the proportion of hesitant parents in the 70%-100% PACV category increased from 9.8% to 13.2%, as seen in Figure 2. This trend outlines a possible link with first-time mothers and the likelihood of vaccine hesitancy.

From a social perspective, Betsch et al. classified the first vaccine as a crucial determinant towards the future conduct of immunization [27]. A mother’s judgement can easily be modified based on the experience that she and her firstborn have with vaccines. Studies have also identified that receiving information during pregnancy may be associated with vaccine uptake [27].

According to the results from this investigation, mothers are more hesitant towards vaccines when compared to fathers. Hesitancy in mothers was 57.4%, whereas the proportion in fathers was 42.6%. The available literature on the topic is controversial. Some investigations concluded that fathers have less lack of confidence in vaccines, especially regarding side effects, when compared to mothers [28], whereas other investigations failed to find this statistically significant association [29]. In general, what can be concluded is that the parent, be it mother or father, plays a relevant role in the vaccine decision-making process.

The proportion of subjects in the 18-29 age group increased from 41.7% to 54.6% when compared to the total subjects. This trend is further supported by the fact that the age group ≥30 years showed an additional decrease in hesitancy behaviors, as seen in Figure 4.

Age, expressed in birth generations, has been closely linked with different perceptions about immunizations. Younger subjects have an increased exposure to social media, which increases the risk and contact with non-academic information [16,22]. Similar studies have confirmed this association between older age and less vaccine hesitancy [28]. Nevertheless, other investigations have failed to provide such association [30].

Some methodologic strengths accompany this investigation. To our knowledge, this study is one of the first investigations to be carried out in the Peruvian context which, to the date of elaboration of this manuscript, had very limited available data on vaccine hesitancy. Furthermore, it confers special importance considering the current SARS-CoV-2 pandemic and the increased attention to vaccines in general. Last, detailed information about vaccine hesitancy can fuel immunization policies, both locally and internationally; clear and evidence-based strategies are needed to make vaccination programs a success.

Yet, there are important limitations that must be considered when interpreting this study. First and foremost, the statistical analysis was designed to show descriptive, non-inferential data representative only of the selected population. Due to the limited number of participants, there was not enough statistical power to execute linear or logistic regression calculations to depict association. This does not allow for extrapolation of the possible causes of the hesitancy. Second, the convenience purposive sampling and telephone interview method are prone to selection and response bias, even if actions were taken to minimize these biases. Finally, it is important to mention the possible confounding factor of the ongoing SARS-CoV-2 pandemic during the execution of this study. Many results could have been fogged due to the urgent, public need of a SARS-CoV-2 vaccine. Further investigation is needed in order to confirm and validate many of the conclusions identified in this research.

## Conclusions

Vaccine hesitancy is a global behavior that is putting millions of lives at risk, especially young and vulnerable individuals. In this study, an important reluctance was identified in the quantity of vaccines administered to the child, as well as potential safety issues and side effects that may arise. Parents’ level of education influenced the hesitancy level, along with the number of children in the household, the relationship to the child, and the age of the parents. The first step to reinstate confidence in immunizations is to understand the true reasons behind this vaccine hesitancy. All healthcare professionals and policymakers should be well informed and conscious about the local factors that feed vaccine uncertainty. By knowing these local factors, key players in Peru will be able to develop mindful plans and effective interventions to strategically tackle this problem.

## References

[1] Andre FE, Booy R, Bock HL (2008). Vaccination greatly reduces disease, disability, death, and inequity worldwide. Bull World Health Organ.

[2] Larson HJ, Jarrett C, Eckersberger E, Smith D, Paterson P (2014). Understanding vaccine hesitancy around vaccines and vaccination from a global perspective: a systematic review of published literature, 2007-2012. Vaccine.

[3] (2021). Report of the SAGE Working Group on Vaccine Hesitancy - October 2014. https://www.who.int/immunization/sage/meetings/2014/october/1_Report_WORKING_GROUP_vaccine_hesitancy_final.pdf.

[4] Vásquez-Uriarte K, Ninatanta Ortiz JA, Romani F, Roque-Henriquez JC (2019). Coverage and factors associated with measles vaccination in children aged 12-59 months in Peru: estimate based on the 2017 Demographic and Family Health Survey. (Article in Spanish). Rev Peru Med Exp Salud Publica.

[5] Opel DJ, Mangione-Smith R, Taylor JA, Korfiatis C, Wiese C, Catz S, Martin DP (2011). Development of a survey to identify vaccine-hesitant parents: the Parent Attitudes about Childhood Vaccines survey. Hum Vaccin.

[6] Opel DJ, Taylor JA, Mangione-Smith R, Solomon C, Zhao C, Catz S, Martin D (2011). Validity and reliability of a survey to identify vaccine-hesitant parents. Vaccine.

[7] Cunningham RM, Kerr GB, Orobio J (2019). Development of a Spanish version of the Parent Attitudes about Childhood Vaccines survey. Hum Vaccin Immunother.

[8] Opel DJ, Taylor JA, Zhou C, Catz S, Myaing M, Mangione-Smith R (2013). The relationship between Parent Attitudes about Childhood Vaccines survey scores and future child immunization status: a validation study. JAMA Pediatr.

[9] Gellin BG, Maibach EW, Marcuse EK (2000). Do parents understand immunizations? A national telephone survey. Pediatrics.

[10] Raof AM (2018). Parental Attitude and beliefs towards child vaccination: Identifying Vaccine Hesitant groups in a family health center, Erbil city Iraq. Mid East J Fam Med.

[11] Groom H, Hopkins DP, Pabst LJ (2015). Immunization information systems to increase vaccination rates: a community guide systematic review. J Public Health Manag Pract.

[12] Opel DJ, Henrikson N, Lepere K, Hawkes R, Zhou C, Dunn J, Taylor JA (2019). Previsit screening for parental vaccine hesitancy: a cluster randomized trial. Pediatrics.

[13] (2021). Multiple vaccinations at once. Multiple Vaccinations at Once | Vaccine Safety | CDC [Internet.

[14] (2021). Vaccines and immunization: myths and misconceptions. https://www.who.int/news-room/q-a-detail/vaccines-and-immunization-myths-and-misconceptions.

[15] Institute of Medicine (US) Immunization Safety Review Committee (2002). Immunization Safety Review: Multiple Immunizations and Immune Dysfunction. https://www.ncbi.nlm.nih.gov/books/NBK220494/.

[16] McKee C, Bohannon K (2016). Exploring the reasons behind parental refusal of vaccines. J Pediatr Pharmacol Ther.

[17] Dubé E, Gagnon D, Nickels E, Jeram S, Schuster M (2014). Mapping vaccine hesitancy - country-specific characteristics of a global phenomenon. Vaccine.

[18] Saada A, Lieu TA, Morain SR, Zikmund-Fisher BJ, Wittenberg E (2015). Parents' choices and rationales for alternative vaccination schedules: a qualitative study. Clin Pediatr.

[19] Taylor LE, Swerdfeger AL, Eslick GD (2014). Vaccines are not associated with autism: an evidence-based meta-analysis of case-control and cohort studies. Vaccine.

[20] García-Fernández L, Hernández AV, Suárez Moreno V, Fiestas F (2013). Addressing the controversy regarding the association between thimerosal-containing vaccines and autism. (Article in Spanish). Rev Peru Med Exp Salud Publica.

[21] Betsch C, Renkewitz F, Betsch T, Ulshöfer C (2010). The influence of vaccine-critical websites on perceiving vaccination risks. J Health Psychol.

[22] Dixon G, Clarke C (2013). The effect of falsely balanced reporting of the autism-vaccine controversy on vaccine safety perceptions and behavioral intentions. Health Educ Res.

[23] Karafillakis E, Dinca I, Apfel F (2016). Vaccine hesitancy among healthcare workers in Europe: a qualitative study. Vaccine.

[24] Lahariya C (2016). Vaccine epidemiology: a review. J Fam Med Prim Care.

[25] Facciolà A, Visalli G, Orlando A (2019). Vaccine hesitancy: an overview on parents’ opinions about vaccination and possible reasons of vaccine refusal. J Public Health Res.

[26] Giambi C, Fabiani M, D’Ancona F (2018). Parental vaccine hesitancy in Italy - results from a national survey. Vaccine.

[27] Betsch C, Bödeker B, Schmid P, Wichmann O (2018). How baby’s first shot determines the development of maternal attitudes towards vaccination. Vaccine.

[28] Ren J, Wagner AL, Zheng A, Sun Z, Boulton ML, Huang Z, Zikmund-Fisher BJ (2018). The demographics of vaccine hesitancy in Shanghai, China. PLoS One.

[29] Alsuwaidi AR, Elbarazi I, Al-Hamad S, Aldhaheri R, Sheek-Hussein M, Hassib N (2020). Vaccine hesitancy and its determinants among Arab parents: a cross-sectional survey in the United Arab Emirates. Hum Vaccin Immunother.

[30] Krishnamoorthy Y, Kannusamy S, Sarveswaran G, Majella MG, Sarkar S, Narayanan V (2019). Factors related to vaccine hesitancy during the implementation of Measles-Rubella campaign 2017 in rural Puducherry - a mixed-method study. J Fam Med Prim Care.

